# Genetic Evidence for a Tight Cooperation of TatB and TatC during Productive Recognition of Twin-Arginine (Tat) Signal Peptides in *Escherichia coli*

**DOI:** 10.1371/journal.pone.0039867

**Published:** 2012-06-26

**Authors:** Frank Lausberg, Stefan Fleckenstein, Peter Kreutzenbeck, Julia Fröbel, Patrick Rose, Matthias Müller, Roland Freudl

**Affiliations:** 1 Institut für Bio- und Geowissenschaften 1, Biotechnologie, Forschungszentrum Jülich GmbH, Jülich, Germany; 2 Institute of Biochemistry and Molecular Biology, Zentrum für Biochemie und Molekulare Zellforschung, University of Freiburg, Freiburg, Germany; 3 Faculty of Biology, University of Freiburg, Freiburg, Germany; Université de Nice-CNRS, France

## Abstract

The twin arginine translocation (Tat) pathway transports folded proteins across the cytoplasmic membrane of bacteria. Tat signal peptides contain a consensus motif (S/T-R-R-X-F-L-K) that is thought to play a crucial role in substrate recognition by the Tat translocase. Replacement of the phenylalanine at the +2 consensus position in the signal peptide of a Tat-specific reporter protein (TorA-MalE) by aspartate blocked export of the corresponding TorA(D^+2^)-MalE precursor, indicating that this mutation prevents a productive binding of the TorA(D^+2^) signal peptide to the Tat translocase. Mutations were identified in the extreme amino-terminal regions of TatB and TatC that synergistically suppressed the export defect of TorA(D^+2^)-MalE when present in pairwise or triple combinations. The observed synergistic suppression activities were even more pronounced in the restoration of membrane translocation of another export-defective precursor, TorA(KQ)-MalE, in which the conserved twin arginine residues had been replaced by lysine-glutamine. Collectively, these findings indicate that the extreme amino-terminal regions of TatB and TatC cooperate tightly during recognition and productive binding of Tat-dependent precursor proteins and, furthermore, that TatB and TatC are both involved in the formation of a specific signal peptide binding site that reaches out as far as the end of the TatB transmembrane segment.

## Introduction

Transport of proteins across biological membranes is catalyzed by membrane-bound transport machineries. In bacteria, the majority of exported proteins are translocated across the cytoplasmic membrane by the general secretion (Sec) translocase which transports its substrates in a more or less unfolded conformation [1], [2]. In addition to the Sec system, many bacteria possess a second protein export system for the translocation of a subset of proteins. Remarkably, this so-called twin-arginine translocation (Tat) system is able to translocate its substrates (often cofactor-containing redox proteins) in a fully folded or even oligomeric state across the cytoplasmic membrane; for recent reviews see [2]–[4].

Both Sec and Tat signal peptides possess a similar tripartite overall structure, comprising a positively-charged amino-terminal region (n-domain), a hydrophobic core (h-domain), and a polar carboxyl-terminal region that contains the recognition site for signal peptidase (c-domain) [5]. However, Tat signal peptides possess a conserved motif (S/T^−1^-R-R-X^+1^-F^+2^-L^+3^-K^+4^; whereby X stands for any amino acid) that is located at the boundary between the n-domain and the hydrophobic h-domain [6], [7]. The importance of this motif for the successful membrane translocation of Tat-dependent precursor proteins has been demonstrated in various site-directed mutagenesis studies [8], [9].

The *Escherichia coli* Tat export apparatus consists of the components TatA/TatE, TatB and TatC [7], [10]. Biochemical analyses from *E. coli* [11] and plant thylakoids [12] have indicated that Tat precursor proteins might contact the Tat components in a hierarchical manner. First, the signal peptide interacts with the primary substrate receptor TatC in a twin-arginine-dependent manner. Subsequently, the signal peptide seems to be transferred to TatB which, in addition to the twin arginine residues, also seems to contact the h-domain of the signal peptide. Finally, the precursor is passed to the translocation pore which, according to most current models, is built up by multiple copies of TatA. No such interactions between precursor and translocase were detected when the twin arginine residues were replaced by a twin lysine pair. Based on these observations, it has been proposed that the initial precursor binding occurs via the recognition of the twin arginine residues by the TatBC receptor complex [11]. Subsequently, this proposal was further strengthened by genetic analyses. In the study by Kreutzenbeck *et* *al.* [13], Tat mutant translocases were identified that were able to suppress the export defect of a TorA(KQ)-MalE hybrid precursor protein in which the twin arginine residues had been replaced by a lysine-glutamine pair. The suppressor mutant translocases possessed single amino acid alterations in either the amino-terminal domains of TatB or TatC. Likewise, mutations in the N-terminal half of TatC were isolated that suppressed the export defect of a TorA-GFP precursor containing a twin lysine pair instead of the twin arginine residues [14]. Both studies corroborated the view that Tat signal peptides are recognized by a receptor complex composed of TatB and TatC and, furthermore, that the twin arginine residues play a crucial role in this recognition process.

Besides the twin arginine residues, other amino acids present in the Tat motif have been shown to act as additional determinants that play a role in the translocation of Tat precursor proteins [9], [15]. It seems a likely possibility that recognition of the Tat signal peptides by the Tat translocase is not solely mediated by the highly conserved twin arginines, but that characteristic properties of the additional amino acid residues, present in the extended Tat motif, also contribute to precursor binding to the TatBC receptor complex.

In the present study, we genetically analyzed the contribution of the +2 position relative to the RR residues in the Tat motif to the specific recognition of Tat signal peptides by the Tat translocase. Replacement of the phenylalanine in the signal peptide of the highly sensitive Tat-specific reporter protein TorA-MalE by an aspartate resulted in a complete export block of the corresponding TorA(D^+2^)-MalE mutant precursor. Suppressor mutations in the Tat components were selected that restored MalE export to various degrees. The suppressing mutations localized to the N-termini of both TatB and TatC. Strikingly, several combinations of TatB and TatC mutations were found to act synergistically in restoring significant export of TorA(D^+2^)-MalE and, interestingly, also of TorA(KQ)-MalE. From these results we conclude that TatB and TatC cooperate tightly in the specific recognition and the productive binding of twin-arginine signal peptides.

## Results

### Replacement of the Phenylalanine at the +2 Position in the Extended Tat Motif by an Aspartate Prevents Tat-dependent export of a TorA-MalE Reporter Protein

The strictly Tat-dependent TorA-MalE reporter protein, consisting of the mature part of the periplasmic maltose-binding protein (MalE) fused to the signal peptide of the periplasmic Tat substrate trimethylamine N-oxide reductase (TorA), allows an easy in situ detection of Tat-dependent MalE export into the periplasm on indicative media (i.e. growth on minimal maltose medium (MMM) and formation of red colonies on MacConkey maltose (MCM) agar plates [13], [16], [17]. As described previously, when plasmid pTorA-MalE is transformed into GSJ101 (a *malE*-negative derivative of the *tat* deletion strain DADE [16], [18], growth on MMM and red colonies on MCM agar plates are only observed when plasmid pHSG-TatABCE (containing the known *tat* genes cloned in an operon-like fashion), but not when the empty vector pHSG575 is cotransformed into the same strain, showing that TorA-MalE export strictly requires the presence of a functional Tat system [13]. Furthermore, as shown in our previous study, the Tat-dependent export of TorA-MalE is completely blocked when the twin arginine (RR) residues in the Tat motif of the TorA signal peptide (S^−1^-R-R-R^+1^-F^+2^-L^+3^-A^+4^) are replaced by a lysine-glutamine (KQ) pair [13].

**Figure 1:**
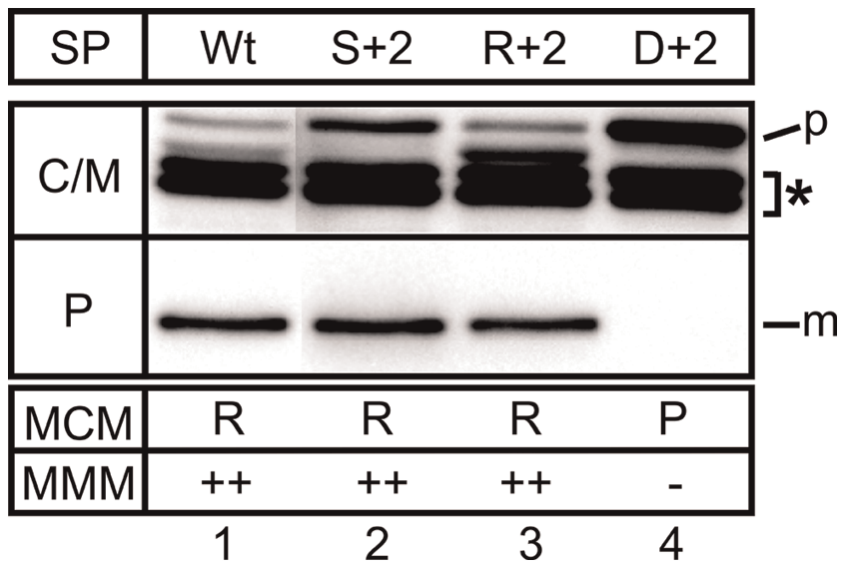
Effect of mutations at the +2 position in the Tat consensus motif. Cells were fractionated into a periplasmic (P) and a combined cytosol/membrane fraction (C/M) by EDTA-lysozyme spheroplasting. The samples were subjected to SDS-PAGE and immunoblotting using anti-MalE antibodies. The positive control was *E. coli* GSJ101 containing plasmids pTorA-MalE and pHSG-TatABCE (lane 1). The other samples correspond to GSJ101 containing plasmid pHSG-TatABCE in addition to pTorA(S^+2^)-MalE (lane 2), pTorA(R^+2^)-MalE (lane 3), or pTorA(D^+2^)-MalE (lane 4). All samples are derived from the same gel. However, some lanes of the gel were removed to make the data easier to interpret. p, precursor protein in the C/M fraction; m, mature MalE in the P fraction; asterisk, TorA-MalE-derived degradation products in the C/M fraction. The phenotypes of the respective strains on MMM (−: no growth; +: slow growth; ++: growth) and MCM (P: pale; LR: light red/pink; R: red) agar plates are shown in the boxes at the bottom of the figure.

To investigate the contribution of amino acid residues in the extended Tat motif other than the nearly invariable RR residues to signal peptide recognition by the Tat translocase, the phenylalanine at position +2 relative to the twin arginine residues was altered to serine, arginine, and aspartate, respectively. Like the wild-type control GSJ101 (pTorA-MalE, pHSG-TatABCE), also GSJ101 (pTorA(S^+2^)-MalE, pHSG-TatABCE) and GSJ101 (pTorA(R^+2^)-MalE, pHSG-TatABCE) were able to grow on MMM and formed red colonies on MCM agar plates, showing that the presence of either a serine or an arginine residue at the +2 position in the Tat motif does not preclude Tat-dependent export of the TorA-MalE reporter. In contrast, no growth on MMM and pale colonies on MCM agar plates were observed with GSJ101 (pTorA(D^+2^)-MalE, pHSG-TatABCE) (Figure 1). The export defect of TorA(D^+2^)-MalE was also directly visualized by determining the subcellular localization of MalE-derived polypeptides after EDTA-lysozyme spheroplasting in the corresponding cells. As shown in Figure 1, lane 1, upper part, several MalE-derived polypeptides are present in the combined fraction of cytosol and membranes (C/M) of GSJ101 (pTorA-MalE, pHSG-TatABCE), coexpressing the wild-type Tat translocase and the unaltered TorA-MalE (positive control). As described previously [17], these bands correspond to the unprocessed precursor protein and a variety of its cyosolic degradation products. In the periplasmic (P) fraction (Figure 1, lane 1, lower part), the mature-sized MalE is detected that has been translocated across the cytoplasmic membrane in a Tat-dependent manner [17]. In full accordance with the in situ phenotypes described above, in both GSJ101 coexpressing the Tat wild-type translocase and TorA(S^+2^)-MalE or TorA(R^+2^)-MalE respectively, mature MalE is present in the P fraction (Figure 1, lanes 2 and 3). In contrast, no mature MalE can be detected in the P fraction of GSJ101 coexpressing the wild-type Tat translocase and TorA(D^+2^)-MalE, showing that the negatively-charged aspartate is not tolerated at the +2 position in the extended twin-arginine motif and renders the TorA signal peptide defective for Tat-dependent protein translocation (Figure 1, lane 4).

### Suppression of the TorA(D^+2^)-MalE Export Defect by KQS Mutant Tat Translocases

In a previous study, we described the identification of Tat mutant translocases (designated KQS for KQ suppressor) that restored substantial export of an otherwise export-defective TorA(KQ)-MalE mutant precursor in which the twin-arginine residues were replaced by a lysine-glutamine pair. Since the KQS suppressor translocases also efficiently accepted the unaltered TorA-MalE precursor, we concluded that the corresponding mutant translocases possess a relaxed specificity with respect to the amino acid residues that occupy the positions of the twin arginine residues in the Tat consensus motif [13]. To analyze whether the KQS mutant translocases could also suppress the export defect caused by the replacement of the F^+2^ by D in the TorA signal peptide, the plasmid encoding either one of the two strongest KQS suppressor translocases, KQS100 (TatC: L9F) or KQS200 (TatB: E8K), was introduced into GSJ101 containing pTorA(D^+2^)-MalE. Subsequently, the resulting strains were tested in situ for MalE export on MMM and MCM indicator plates and on the protein level by cell fraction experiments. GSJ101 expressing the export defective TorA(D^+2^)-MalE reporter together with the KQS100 (TatC: L9F) mutant translocase could not grow on MMM plates and formed pale colonies on MCM. Furthermore, no mature MalE was found in the P fraction of the corresponding cells (Figure S1, lane 4). Taken together, these data show that the strongest KQ suppressor [13] is not able to suppress the export defect caused by the F→D alteration at the +2 position in the extended Tat motif to a significant degree. Strikingly and in sharp contrast, GSJ101 expressing the KQS200 (TatB: E8K) mutant translocase together with TorA(D^+2^)-MalE showed growth on MMM and formed red colonies on MCM. In addition, a low but significant amount of mature MalE is present in the P fraction of the corresponding cells (Figure S1, lane 3). These latter results indicate that, although originally selected against a totally different mutation in the Tat consensus motif (i.e. RR→KQ [13]), the mutation E8K in TatB results in a Tat mutant translocase that can also suppress the export defect of a TorA(D^+2^)-MalE mutant precursor.

### Selection for Mutant Tat Translocases that Suppress the Export Defect of the TorA(D^+2^)-MalE Precursor Protein

Next, we asked whether new Tat mutant translocases could be isolated that can productively recognize and translocate the export defective TorA(D^+2^)-MalE mutant precursor. As the starting point for our mutagenesis, we used the *tatABCE* genes encoding the KQS100 (TatC: L9F) mutant translocase. As described in the previous section, the L9F mutation in TatC did not confer significant export to the TorA(D^+2^)-MalE precursor. Nevertheless, we thought that it might be possible to select for additional mutations that, alone or in combination with the L9F mutation in TatC, allow for the suppression of the TorA(D^+2^)-MalE export defect.

Using pHSG-TatABCE-KQS100 (Table 1, [13]) as a template, the *tatABCE* genes were mutagenized by error-prone (ep)-PCR. The corresponding *tatABCE* ep-PCR fragments were cloned into the low-copy vector pHSG575 and the resulting pool of mutagenized pHSG-TatABCE plasmids was transformed into GSJ101 (pTorA(D^+2^)-MalE) and subsequently plated onto MMM agar plates. After two days of incubation, the formation of single colonies was observed. Three randomly chosen colonies that, after re-streaking, showed reproducible growth were selected for further characterization.

**Table 1:** Bacterial strains and plasmids used in this study.

Strains or plasmids	Relevant properties^a^	Source
***E.coli*** ** strains**		
XL1-Blue	*recA1 endA1 gyrA96 thi-1 hsdR17 supE44 relA1 lac(F’ proAB lacIqZΔM15 Tn10 (*Tc^R^ *))*	Stratagene
GSJ100	MC4100×P1.MM129>Tc^R^ ΔmalE444 *zjb*729::Tn*10*	[16]
GSJ101	DADE×P1.MM129>Tc^R^ ΔmalE444 *zjb*729::Tn*10*	[16]
**Plasmids**		
pHSG575	pSC101 replicon, *lac*Zα+ Cm^R^	[44]
pHSG-TatABCE	pHSG575 derivative; carring the *tatABCE* genes of *E. coli*	[17]
pHSG-TatABCE-RRD1	pHSG-TatA(D58G)B(L9P, N119K, T133M) C(L9F, R19C, M159T)E(Q59R)	This study
pHSG-TatABCE-RRD1	pHSG-TatA(D58G)B(L9P, N119K, T133M) C(L9F, R19C, M159T)E(Q59R)	This study
pHSG-TatABCE-RRD2	pHSG-TatAB(P152R)C(L9F, K18E, L34Q, F118L, V167A)E	This study
pHSG-TatABCE-RRD3	pHSG-TatAB(L9Q)C(L9F, M163L)E	This study
pHSG-TatABCE-RRD5	pHSG-TatAB(L9Q)C(L9F, K18E)E	This study
pHSG-TatABCE-RRD6	pHSG-TatAB(L9Q)C(K18E)E	This study
pHSG-TatABCE-KQS100	pHSG-TatABC(L9F)E	[13]
pHSG-TatABCE-KQS105	pHSG-TatABC(K18E)E	[13]
pHSG-TatABCE-KQS200	pHSG-TatAB(E8K)CE	[13]
pHSG-TatABCE-RRD1-1	pHSG-TatAB(L9P)CE	This study
pHSG-TatABCE-RRD1-3	pHSG-TatAB(L9P)C(L9F)E	This study
pHSG-TatABCE-RRD2-3	pHSG-TatABC(L9F, K18E)E	This study
pHSG-TatABCE-RRD3-1	pHSG-TatAB(L9Q)CE	This study
pHSG-TatABCE-RRD3-3	pHSG-TatAB(L9Q)C(L9F)E	This study
pTorA-MalE	pBBR1MCS-2 carrying the *torA-malE* fusion gene, Km^R^	[16]
pTorA(KQ)-MalE	pTorA-MalE (R11K, R12Q)	[13]
pTorA(D^+2^)-MalE	pTorA-MalE (F14D)	This study
pTorA(S^+2^)-MalE	pTorA-MalE (F14S)	This study
pTorA(R^+2^)-MalE	pTorA-MalE (F14R)	This study
pCGTorA-GFP	*E. coli/Corynebacterium glutamicum* shuttle vector containing the *torA-gfp* fusion gene	[40]
pTorA-GFP	pBBR1MCS-2 carrying the *torA-gfp* fusion gene, Km^R^	This study
pTorA(D^+2^)-GFP	pTorA-GFP (F14D)	This study

a Km^R^, kanamycin resistance; Cm^R^, chloramphenicol resistance; Tc^R^, tetracycline resistance.

**Figure 2:**
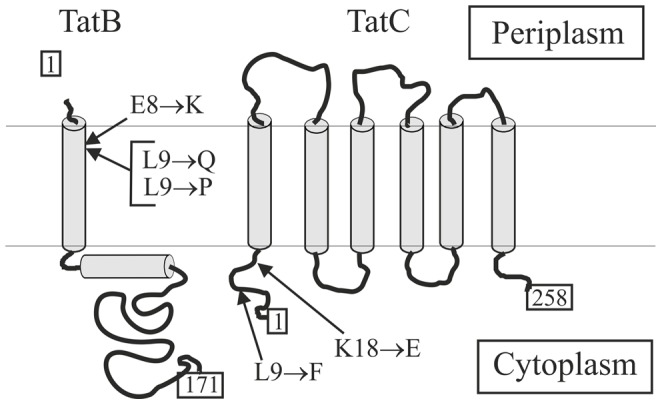
Membrane topology of *E. coli* TatB and TatC and positions of mutations. Arrows indicate the positions of mutations that are involved in the suppression of the TorA(D^+2^)-MalE and TorA(KQ)-MalE export defects.

DNA sequencing of the corresponding plasmids (pHSG-TatABCE-RRD1 to 3) showed that, in addition to the L9F mutation in TatC, multiple mutations are present in the respective mutant Tat translocases (Table 2). Interestingly, similar to the previously described KQS mutant Tat translocases that allowed export of the TorA(KQ)-MalE precursor [13], in each of the mutant translocases (designated RRD1 to 3), some of the newly selected mutations present in the multiple mutants map to the extreme amino-terminal ends of TatB and TatC (Table 2, Figure 2). Subsequently, these mutations were analyzed with respect to their contribution to the suppressing activity by introducing them alone or in pairwise combinations into otherwise wild-type *tat* genes (Table 2, and see below). Furthermore, the amounts of TatA, TatB and TatC proteins, present in the membrane fractions of the strains expressing these specifically constructed Tat translocases, were analyzed by Western blotting (Figure S2A). In most cases, the amounts of the Tat components were found to be similar or somewhat lower compared to the wild-type control. A noticeable difference, however, was found for TatB containing the mutation L9P. Here, the mutant TatB protein was only detected in the membrane after over-exposure of the Western blot (Figure S2B), suggesting that the respective TatB protein is relatively unstable and proteolytically degraded. Nevertheless, the mutant TatB protein is unequivocally required for the suppressing activity of the corresponding mutant translocases, since a Tat translocase lacking TatB did not show export of the TorA(D^+2^)-MalE mutant precursor (data not shown). Importantly, however, these findings exclude the possibility that the observed gain-of-function phenotypes, conferred by the mutant Tat translocases, are simply due to increased amounts of one or more Tat proteins and are in fact caused by the corresponding mutations.

**Table 2:** Amino acid alterations present in mutant translocases.

Mutant translocase	Amino acid alterations
**KQS100**	TatC: L9→F
**KQS105**	TatC: K18→E
**KQS200**	TatB: E8→K
**RRD1**	TatA: D58→G
	TatB: **L9→P**, N119→K, T133→M
	TatC: **L9→F**, R19→C, M159→T
	TatE: Q59→R
**RRD1-1**	TatB: L9→P
**RRD1-3**	TatB: L9→P
	TatC: L9→F
**RRD2**	TatB: P152→R
	TatC: **L9→F**, **K18→E**, L34→Q, F118→L, V167→A
**RRD2-3**	TatC: L9→F, K18→E
**RRD3**	TatB: **L9→Q**
	TatC: **L9→F**, M163→L
**RRD3-1**	TatB: L9→Q
**RRD3-3**	TatB: L9→Q
	TatC: L9→F
**RRD5**	TatB: L9→Q
	TatC: L9→F, K18→E
**RRD6**	TatB: L9→Q
	TatC: K18→E

Mutations present in the multiple mutants RRD1, RRD2, and RRD3 that are mainly responsible for the suppression of the TorA(D^+2^)-MalE and TorA(KQ)-MalE export defects are indicated in bold.

### Identification of the Mutations in the Multiple RRD Mutant Tat Translocases that are Responsible for Restoring Membrane Translocation of the Export-defective TorA(D^+2^)-MalE Precursor

**Figure 3:**
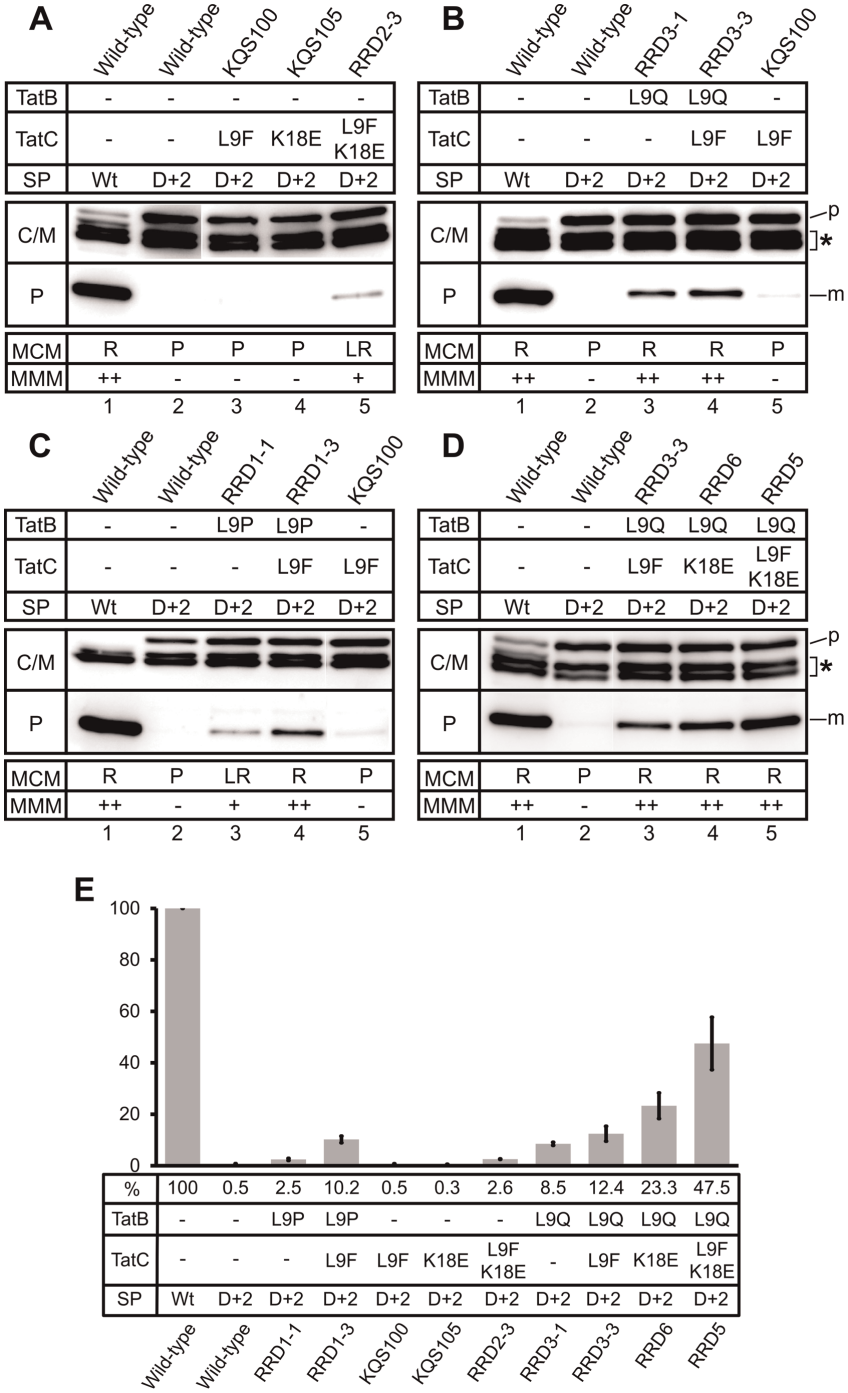
Subcellular localization of TorA(D^+2^)-MalE-derived polypeptides. Cells were fractionated into a periplasmic (P) and a combined cytosol/membrane fraction (C/M) by EDTA-lysozyme spheroplasting. The samples were subjected to SDS-PAGE and immunoblotting using anti-MalE antibodies. The positive control was *E. coli* GSJ101 containing plasmids pTorA-MalE and pHSG-TatABCE (lanes 1). All other samples correspond to GSJ101 containing plasmid pTorA(D^+2^)-MalE in addition to a pHSG-TatABCE plasmid that encodes one of the mutant translocases derived from RRD2 (**A**), RRD3 (**B**), RRD1 (**C**), or the synthetic mutant translocases RRD5/6 (**D**), as indicated above the lanes. p, TorA-MalE/TorA(D^+2^)-MalE precursor in the C/M fraction; m, mature MalE in the P fraction; asterisk, TorA-MalE/TorA(D^+2^)-MalE degradation products in the C/M fraction. All samples shown in the respective panels are derived from the same gel. However, in some cases lanes of the gels were removed to make the data easier to interpret. The phenotypes of the respective strains on MMM (-: no growth; +: slow growth; ++: growth) and MCM (P: pale; LR: light red/pink; R: red) agar plates are shown in the boxes at the bottom of the figure. The nature of the signal peptide (SP) of the respective TorA-MalE precursors (wild-type (Wt) or containing the D^+2^ mutation (D+2)) and the TatB and/or TatC mutations present in the respective translocases are indicated in the boxes at the top of the panels. **E**. Relative export efficiencies. The amount of MalE in the P fraction of strains expressing TorA-MalE or TorA(D^+2^)-MalE in combination with the Tat translocases indicated below the bars was determined in at least three independent experiments via quantification of the chemiluminescence signals. The signals were recorded by a CCD camera and subsequently analyzed by the program AIDA 4.15 (Raytest). %, average value of relative export efficiency. The relative export efficiency of the respective positive control strain GSJ101 (pTorA-MalE, pHSG-TatABCE) in each experiment (lane 1 in panels A–D) was set to 100%.

TorA(D^+2^)-MalE export in the strains expressing the various Tat mutant translocases was analyzed indirectly by MMM and MCM plate assays and directly by determining the amount of MalE in the periplasm (Figure 3). The relative export efficiency (reflected by the amount of mature-sized MalE present in the periplasm) of the positive control strain, to which all further relative export efficiencies described in this work will be related, was set to 100%. All numbers indicated in the following represent average relative export efficiencies obtained from at least three independent experiments. From these combined analyses, it became evident that in all three mutant isolates a synergistically acting combination of two mutations (i.e. the L9F mutation in TatC together with a newly selected mutation in either TatC or TatB) is responsible for the suppression of the TorA(D^+2^)-MalE export defect.

In suppressor mutant translocase RRD2, two synergistically acting amino acid alterations (L9F and K18E) both located in the extreme amino-terminal domain of TatC are responsible for suppressing the TorA(D^+2^)-MalE export defect. Interestingly, similar to the L9F mutation in TatC, the additionally selected K18E mutation in TatC had also been identified previously as a mutation that can suppress the export defect of TorA(KQ)-MalE (mutant translocase KQS105 [13]). The RRD2-derived mutant translocase RRD2-3 (TatC: L9F, K18E) showed comparable growth on MMM and the same formation of light red (pink) colonies on MCM agar plates as the original RRD2 isolate (data not shown). In contrast, neither of the mutations alone allowed significant export of TorA(D^+2^)-MalE, since strains containing either one of the mutant translocases KQS100 (TatC: L9F) or KQS105 (TatC: K18E) did not grow on MMM and formed pale colonies on MCM agar plates (Figure 3A). The amount of exported TorA(D^+2^)-MalE in the periplasm of the respective RRD2-derived strains perfectly reflects their phenotypic behavior observed in the plate assays. As shown in Figure 3A and 3E, hardly any mature MalE protein can be detected in the periplasmic fractions of GSJ101 coexpressing TorA(D^+2^)-MalE and the mutant translocases KQS100 (TatC: L9F) or KQS105 (TatC: K18E). In contrast, low but nevertheless significant amounts of mature MalE are present in the periplasmic fraction of GSJ101 expressing the double mutant Tat translocase RRD2-3 (TatC: L9F, K18E), corresponding to a relative export efficiency of 2.6%.

In contrast to mutant RRD2, a single mutation located in the extreme amino-terminal domain of TatB together with the L9F mutation in TatC were found to synergistically contribute to the suppression of the TorA(D^+2^)-MalE export defect in the mutants RRD1 and RRD3. In mutant RRD3, the mutations L9Q in TatB and L9F in TatC were found to be responsible for the suppressing activity. The mutant translocase RRD3-1 (TatB: L9Q) already showed a strong suppressing activity in the in situ plate assays (growth on MMM; red colonies on MCM; Figure 3B). As shown in Figure 3B and 3E, a low synergistic effect of the two mutations can be seen when the relative export efficiencies of the TorA(D^+2^)-MalE precursor are compared with values of 8.5% observed for RRD3-1 (TatB: L9Q), 0.5% for KQS100 (TatC: L9F), and 12.4% for the double mutant RRD3-3 (TatB: L9Q; TatC: L9F), indicating that the additional presence of the TatC (L9F) mutation seems to further enhance the suppressing effect of the TatB (L9Q) mutation. Nevertheless, the difference in the export efficiencies for RRD3-1 and RRD3-3 is very small for the TorA(D^+2^)-MalE precursor and, due to the semiquantitative nature of our method, a solid conclusion with respect to a synergy between the two mutations might be premature at this point. However, as shown below, the synergy between the two mutations becomes very clear when the export of another defective precursor (TorA(KQ)-MalE) in combination with the mutant translocases is analyzed.

In mutant RRD1, a more pronounced synergy between a TatB mutation (L9P) and the L9F mutation in TatC was found to be responsible for the suppression of the TorA(D^+2^)-MalE export defect. As shown in Figure 3C, the single TatB mutation (RRD1-1 (TatB: L9P)) alone showed a low but significant suppressing activity in the in situ plate assays (i.e. slow growth on MMM; light red (pink) colonies on MCM). In contrast, the combination with the KQS100 (TatC: L9F) mutation, which by itself does not possess any significant suppressing activity (i.e. no growth on MMM; pale colonies on MCM), in the double mutant translocase RRD1-3 (TatB: L9P; TatC: L9F) promoted a clearly stronger suppression phenotype in the plate assays (i.e. growth on MMM and red colonies on MCM) of GSJ101 expressing TorA(D^+2^)-MalE. The corresponding relative export efficiencies of the TorA(D^+2^)-MalE precursor paralleled the in situ phenotypes, with values of 2.5% observed for RRD1-1 (TatB: L9P), 0.5% for KQS100 (TatC: L9F), and, strikingly, 10.2% for the double mutant RRD1-3 (TatB: L9P; TatC: L9F), clearly showing the synergistic action of the TatB (L9P) and the TatC (L9F) mutations (Figure 3C and 3E).

### Combinations of TatB and TatC Mutations that Further Enhance TorA(D^+2^)-MalE Export

Next, suppressor mutations derived from different RRD isolates were combined in the synthetic double mutant translocase RRD6 (TatB: L9Q; TatC: K18E) and in the triple mutant translocase RRD5 (TatB: L9Q; TatC: L9F, K18E) and export of TorA(D^+2^)-MalE conferred by these artificially created translocases was analyzed on indicator plates and directly by cell fractionation experiments (Figure 3D and 3E). GSJ101 expressing TorA(D^+2^)-MalE together with RRD6 (TatB: L9Q; TatC: K18E) showed growth on MMM, a formation of red colonies on MCM, and a relative export efficiency of 23.3%. When compared to the export efficiencies obtained with the single mutant translocases RRD3-1 (TatB:L9Q) (8.5%) and KQS105 (TatC: K18E) (0.3%) described in the previous section, the RRD6 double mutant translocase was found to be significantly more active with respect to TorA(D^+2^)-MalE export. An even further increase in the suppressing activity was observed when the KQS100 (TatC: L9F) mutation was added to the two RRD6 mutations. The presence of the resulting triple mutant translocase RRD5 (TatB: L9Q, TatC: L9F, K18E) in GSJ101 expressing TorA(D^+2^)-MalE resulted in a significant further increase of the relative export efficiency of TorA(D^+2^)-MalE (i.e. 47.5%) compared to the corresponding RRD6 double mutant translocase. These results clearly demonstrate that the absent or small effects that each mutation exerts in a single context do combine to a very strong effect in the RRD6 double mutant translocase, and an even stronger effect in the RRD5 triple mutant translocase.

### The Synergistic Combinations of TorA(D^+2^)-MalE Suppressor Mutations in the TatBC Receptor Complex also Synergistically Suppress the Export Defect of a TorA(KQ)-MalE Precursor Protein

**Figure 4:**
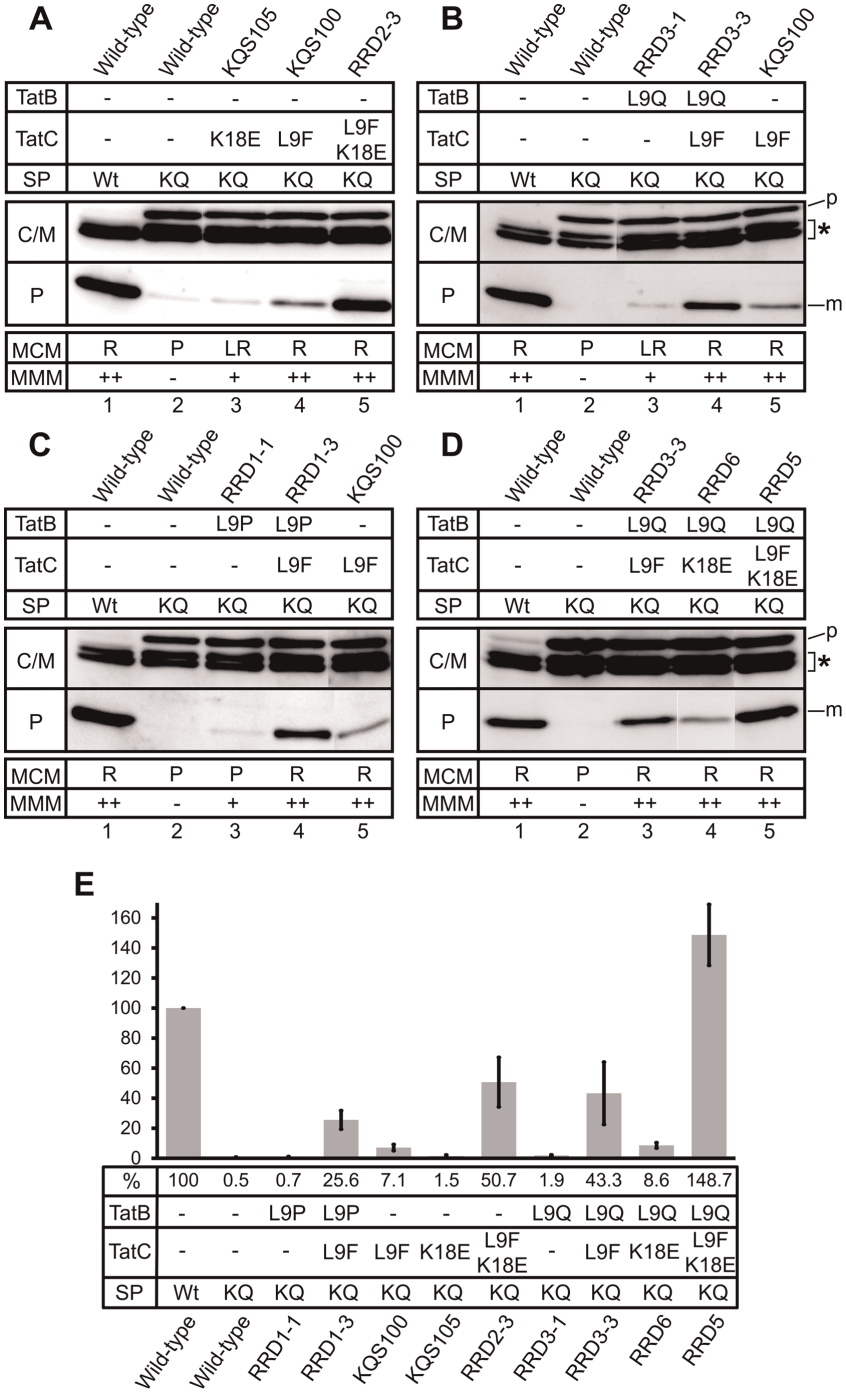
Subcellular localization of TorA(KQ)-MalE-derived polypeptides. Cells were fractionated into a periplasmic (P) and a combined cytosol/membrane fraction (C/M) by EDTA-lysozyme spheroplasting. The samples were subjected to SDS-PAGE and immunoblotting using anti-MalE antibodies. The positive control was *E. coli* GSJ101 containing plasmids pTorA-MalE and pHSG-TatABCE (lanes 1). All other samples correspond to GSJ101 containing plasmid pTorA(KQ)-MalE in addition to a pHSG-TatABCE plasmid that encodes one of the mutant translocases derived from RRD2 (**A**), RRD3 (**B**), RRD1 (**C**), or the synthetic mutant translocases RRD5/6 (**D**), as indicated above the lanes. p, TorA-MalE/TorA(KQ)-MalE precursor in the C/M fraction; m, mature MalE in the P fraction; asterisk, TorA-MalE/TorA(KQ)-MaE degradation products in the C/M fraction. All samples shown in the respective panels are derived from the same gel. However, in some cases lanes of the gels were removed to make the data easier to interpret. The phenotypes of the respective strains on MMM (-: no growth; +: slow growth; ++: growth) and MCM (P: pale; LR: light red/pink; R: red) agar plates are shown in the boxes at the bottom of the figure. The nature of the signal peptide (SP) of the respective TorA-MalE precursors (wild-type (Wt) or containing the KQ mutation (KQ)) and the TatB and/or TatC mutations present in the respective translocases are indicated in the boxes at the top of the panels. **E**. Relative export efficiencies. The amount of MalE in the P fraction of strains expressing TorA-MalE or TorA(KQ)-MalE in combination with the Tat translocases indicated below the bars was determined in at least three independent experiments via quantification of the chemiluminescence signals. The signals were recorded by a CCD camera and subsequently analyzed by the program AIDA 4.15 (Raytest). %, average value of relative export efficiency. The relative export efficiency of the respective positive control strain GSJ101 (pTorA-MalE, pHSG-TatABCE) in each experiment (lane 1 in panels A–D) was set to 100%.

Subsequently, we analyzed whether the combinations of Tat mutations that synergistically suppress the export defect of TorA(D^+2^)-MalE likewise showed a synergistic suppression of the export defect of the TorA(KQ)-MalE precursor. As described previously, the presence of the L9F mutation in TatC (mutant translocase KQS100) already allows significant export of TorA(KQ)-MalE to the periplasm [13]. The corresponding strain GSJ101 (pTorA(KQ)-MalE, pHSG-TatABCE-KQS100) showed growth on MMM and red colonies on MCM agar plates (Figure 4). Identical results (i.e. growth on MMM and red colonies on MCM) in these in situ plate assays were observed for GSJ101 expressing the TorA(KQ)-MalE precursor together with the Tat translocases containing the TatC: L9F mutation in combination with one (or two) of the newly selected TatB or TatC mutations (Figure 4). These findings already indicate that the additional mutations do not exert a negative effect on the suppression activity of the TatC: L9F mutation. In contrast, the subsequent cell fractionation experiments revealed that, in all cases, the additional mutations even confer a striking increase in the relative export efficiencies of TorA(KQ)-MalE when compared to the efficiency (7.1%) conferred by the KQS100 (TatC:L9F) mutation alone (Figure 4).

Whereas the single mutant translocase KQS105 (TatC:K18E) promoted only relatively low levels of TorA(KQ)-MalE export to the periplasm (1,5%), the combination of the two TatC mutations in RRD2-3 (TatC: L9F, K18E) resulted in a significant increase in the relative TorA(KQ)-MalE export efficiency (50.7%), demonstrating that, compared to the results for the TorA(D^+2^)-MalE precursor, the synergistic effect of the two TatC mutations is much more pronounced for the suppression of the TorA(KQ)-MalE export defect (Figure 4A and 4E).

Similar results were obtained for combinations of the TatC: L9F or the TatC: K18E mutation with mutations TatB: L9Q or TatB: L9P (Figure 4B,C,D,E). Whereas only low levels of TorA(KQ)-MalE export were promoted by the single mutant translocases RRD3-1 (TatB: L9Q) and RRD1-1 (TatB: L9P), resulting in relative export efficiencies of 1.9% and 0.7% respectively, a strong increase in the relative TorA(KQ)-MalE export efficiencies was found for GSJ101 expressing the corresponding double mutant translocases RRD3-3 (TatC: L9F; TatB: L9Q) (43.3%), RRD1-3 (TatC: L9F; TatB: L9P) (25.6%), and RRD6 (TatC: K18E; TatB: L9Q) (8.6%). An even further increased relative TorA(KQ)-MalE export efficiency (148.7%) was observed for the triple mutant translocase RRD5 (TatC: L9F, K18E; TatB: L9Q). Importantly, the relative export efficiencies observed for the double- and also the triple mutant translocases were significantly higher than the sum of the export efficiencies observed for the respective single mutant translocases, clearly demonstrating that the combined mutations in TatB and TatC synergistically act together in promoting export of the normally completely export-defective TorA(KQ)-MalE precursor.

## Discussion

The Tat consensus motif is a short stretch of amino acids (S/T-R-R-X-F-L-K) that is located at the boundary between the n- and the h-region of Tat signal peptides and is thought to play an important role in the specific recognition of Tat substrates by the Tat translocase [11], [13], [19], [20]. Clearly, the nearly invariant two arginine residues are the most important contributing factor, since even a conservative replacement of RR by KK results in significantly reduced export efficiencies or even a complete export block; e.g. [13], [21], [22]. A somewhat greater flexibility seems to exist with respect to the amino acids that can be tolerated at positions −1, +2, and +3 relative to the RR residues. Here, even non-conservative amino acid changes at the respective positions do often still allow significant export, although even at these positions, not every amino acid is tolerated [9], [15], [22]. Notably, the effect of an identical mutation can vary somewhat, depending on the precursor and the sensitivity of the corresponding export assay under study. Using MalE as a very sensitive export reporter, we now showed that replacing the phenylalanine at position +2 in the TorA signal peptide by either a serine (S^+2^) or even an arginine (R^+2^) did not prevent membrane translocation of the corresponding TorA(S^+2^)-MalE or TorA(R^+2^)-MalE precursor proteins. In contrast, export of a TorA(D^+2^)-MalE precursor, in which the phenylalanine was replaced by an aspartate (D^+2^), was completely blocked. The latter result indicates that the presence of an aspartate at the +2 position in the Tat motif interferes with the recognition and/or productive binding of the TorA signal peptide by the Tat translocase.

The export defect of TorA(D^+2^)-MalE could be significantly suppressed by a mutation (TatB: E8K), that was previously isolated as a suppressor mutation for the export defect of TorA(KQ)-MalE [13]. Since also the unaltered TorA-MalE precursor was efficiently accepted by the respective KQS200 (TatB: E8K) mutant translocase [13], these combined results indicate that the E8K mutation in TatB results in a relaxed specificity of signal peptide recognition by the Tat translocase. A possible explanation for such a relaxation might be that the TatB: E8K mutation results in a conformational alteration of a signal peptide binding pocket of the translocase in such a way that missing binding contacts between the main specificity determinants of the signal peptide (i.e. the amino acids of the Tat consensus motif) and the binding pocket are compensated by stronger contacts between the binding pocket and one or more regions located elsewhere in the signal peptide. In contrast, the strongest TorA(KQ)-MalE suppressor mutant translocase KQS100 (TatC: L9F) isolated in our previous study did not significantly suppress the export defect of TorA(D^+2^)-MalE, indicating that not all combinations of precursor proteins and altered Tat translocases result in a functional interaction between the partners involved.

To allow a further genetic analysis of signal peptide recognition by the Tat translocase and to identify the partners and the regions within these partners that are involved in the substrate binding event, we selected for mutant Tat translocases that are able to restore export of the TorA(D^+2^)-MalE precursor. To do so, we randomly mutagenized the Tat translocase KQS100 by epPCR, aiming at mutations that, together with the TatC: L9F mutation which by itself does not promote any significant TorA(D^+2^)-MalE export, do restore a productive interaction of TorA(D^+2^)-MalE with the Tat substrate receptor site and allow Tat-specific translocation of MalE into the periplasm. In fact, it was possible to identify three different point mutations located either in TatC (K18E) or in TatB (L9P or L9Q) that showed clear synergies when paired with the L9F mutation in TatC in restoring export of TorA(D^+2^)-MalE and, interestingly, even more pronounced also of the TorA(KQ)-MalE precursor. The three mutations are located in the same regions as the mutations found in our previously isolated KQS mutant translocases [13], namely in the amino-terminal regions of TatB and TatC, respectively, adding further evidence for an involvement of both regions in the binding of Tat signal peptides. Interestingly, recently reported biochemical data directly showed that both regions indeed come into close contact with precursor proteins prior to their translocation. A systematic crosslinking approach where a photoreactive amino acid was incorporated at various positions of TatC and TatB, respectively, revealed that contacts exist between Tat signal peptides and almost the entire cytosolic N-terminus (spanning amino acid residues 3 to 20) of TatC [23]. In addition, crosslinks between position 9 of TatB and Tat precursor proteins were obtained, indicating that the precursors must have been bound to the TatBC receptor such that they come into close proximity even to the periplasmic end of the TatB transmembrane helix [24]. Our TatB suppressor mutations affecting position 8 (mutation E8K [13]) and position 9 (mutations L9P and L9Q identified in this study) are, according to the topological model of TatB, located in the periplasmically oriented end of the transmembrane domain. Therefore, their effects must either be due to long-range conformational effects that are transmitted via the transmembrane segment to a binding pocket located at the cytoplasmically oriented side of the TatBC receptor or, in line with the recent cross-linking data [23], [24], affect the binding of the precursor at a stage where the signal peptide and the early mature part of the precursor has been transferred into an advanced-stage precursor binding site that reaches out as far as the periplasmically oriented end of the transmembrane helix of TatB. Importantly, the observed synergies between mutations in TatC (L9F; K18E) and mutations located in TatB (L9P; L9Q) now add strong genetic evidence for a tight cooperation of TatB and TatC during signal peptide recognition and, very likely, for the involvement of both components in the formation of a specific signal peptide binding site.

As an attempt to reconcile previously published data with the results of our present study, we would like to propose the following model: Tat-dependent precursor proteins approach the TatBC receptor complex either directly from the cytosol or, as suggested by various recent reports [25]–[27], via a membrane-lipid associated form. After initial binding of the precursor to TatBC, which might not critically depend on the presence of the twin-arginines in the Tat consensus [28]–[30], the precursor is subsequently threaded deep into the TatBC receptor complex, reaching out as far as the periplasmic end of the TatB transmembrane helix. In this advanced state of precursor binding to the translocase, most likely a hairpin loop is formed that consists of the signal peptide and the early mature region of the precursor. In fact, experimental evidence for a loop-insertion mechanism [31] and, likewise, for a state in which the signal peptide is deeply inserted into the translocase [32] has been obtained for the thylakoidal Tat system. Based on the location and, most importantly, on the synergistic behavior of our suppressor mutations, we propose that the N-terminal regions of both TatB and TatC are part of the advanced-stage precursor binding site and that both regions cooperate in the specific and productive binding of Tat signal peptides. For a successful binding event, most likely several important docking contacts are required between the signal peptide and the surface of the binding site. In this process, the amino acids present in the Tat consensus motif of the signal peptide are very likely major contributing factors to the binding specificity and, furthermore, to the overall binding affinity. Following a productive binding event, the precursor is ready for its subsequent translocation across the membrane.

Replacement of crucial positions in the Tat consensus motif by other amino acids can result in an export defect of the respective precursor proteins (such as e.g. TorA(KQ)-MalE or TorA(D^+2^)-MalE). We propose that this, besides possible sterical problems that cannot be excluded, is due to a reduced overall binding affinity of the precursor to the signal peptide binding site as a consequence of the lack of important binding contacts between the altered signal peptide and the binding site. As a consequence, the respective precursor proteins might not bind tight or long enough to the signal peptide binding site to allow their subsequent translocation and, therefore, are rapidly released back from the TatBC receptor. Interestingly, a recent report has provided evidence that translocon-bound precursor proteins can bind and dissociate from the translocon on a relatively rapid time scale and that a translocon interaction may only occasionally result in a translocation event [27]. In the light of these results, the proposed weakening of the overall affinity of the precursor to the advanced-stage binding site in the TatBC receptor complex by mutations in the Tat consensus motif is expected to decrease (or in the extreme case to completely prevent) its chance of being translocated.

As described in the present work, mutations in TatB and TatC can be identified that can significantly restore export of both the TorA(KQ)-MalE and the TorA(D^+2^)-MalE precursor proteins. Furthermore, the corresponding mutant translocases are still able to handle the unaltered TorA-MalE precursor (Figure S3), clearly showing that the suppressor mutations do not act in a strictly allele-specific manner. Furthermore, since the behavior of the suppressor translocases is very similar also with respect to the suppression of the export defect of a TorA(D^+2^)-GFP (green fluorescence protein) reporter (Figure S4), it is very likely that the suppressor mutations in fact influence the recognition of the signal peptide, rather than that of the mature part of the respective precursor protein. Taken together, these findings strongly suggest that, in the case of our export-defective mutant precursors, the weakened or lacking binding contacts between the Tat consensus motif residues and the precursor binding site are most likely compensated by increased binding contacts between the binding site and (so far unknown) amino acids elsewhere in the signal peptide. Our finding that the effects of the single mutations more than sum up with respect to their suppression efficiency when the mutations are combined in double or triple mutant translocases strongly suggests that each of the mutations causes a subtle alteration of the advanced stage precursor binding site that, when combined, results in an optimized binding groove for even normally completely export-defective Tat precursor proteins.

Recent studies have shown that two or even four precursors can simultaneously bind to a Tat translocase [33], [34], whereby each precursor seems to be bound to an isolated binding site [34]. Although the exact nature of the advanced-stage precursor binding site proposed in this study and the number of TatB and TatC protomers that are involved in its formation are presently unknown, our results provide clear genetic evidence for an involvement of the amino-terminal regions of both TatB and TatC in the formation of such an advanced-stage binding site and, furthermore, for a close cooperation of TatB and TatC during the specific recognition and binding of Tat-dependent precursor proteins at a discrete and decisive step prior to the subsequent actual membrane translocation event.

Signal peptide binding sites of protein transport components have been characterized in atomic resolution details in other systems. For example, the h-regions of signal peptides of signal recognition particle (SRP)-dependent substrates bind in an α-helical conformation to helices that line a hydrophobic binding groove within the M-domain of the SRP54/Ffh protein by a 4–4 "ridges-into-grooves" helix packing. Furthermore, this binding seems to involve a conformational change within SRP54/Ffh, indicating that the binding event probably involves an induced fit mechanism to maximize the hydrophobic interactions with particular signal peptides [35]. Another example is the signal peptide binding site within the SecYEG pore complex. Here, upon of insertion of the signal peptide into SecYEG, it intercalates into the lateral gate of SecY that is formed by transmembrane helices TM2a, TM3, TM7, and TM8. In its bound form, the h-region of the signal peptide forms a helix of approximately two turns which is thought to be accommodated in a window in the lateral gate of SecY [36]. In contrast, no comparable details are known so far for the binding of Tat signal peptides to the TatBC receptor complex. In analogy to the systems described above, a possible way to interpret our genetic data is that, during an advanced stage binding step, an intercalation of Tat precursor proteins occurs between TatB and TatC transmembrane helices for which, also in the case of Tat, the h-region of the signal peptide might play an important role. However, since conformational effects affecting even distantly located parts or other functions of TatBC besides precursor recognition cannot be completely excluded so far for any of our suppressor mutations, their proposed mode of action that we have suggested in our model should be taken as that what it is meant to be, namely a working hypothesis that has to be proven or disproven by further experimentation.

## Materials and Methods

### Bacterial Strains, Plasmids, and Culture Conditions

The bacterial strains and plasmids used in this study are listed in Table 1. Bacterial strains were grown at 37°C in Luria Bertani medium [37], minimal medium [38] supplemented with 0.4% maltose, or MacConkey agar base medium (Difco) supplemented with 1% maltose. If required, isopropyl-β-D-thiogalactopyranoside was used in a 0.1 mM concentration. Antibiotic supplements were used in the following concentrations: kanamycin, 50 mg/l; chloramphenicol, 25 mg/l; and tetracycline, 15 mg/l.

### DNA Manipulations

All DNA manipulations followed standard procedures [39]. Oligonucleotides used as PCR primers are listed in Supplementary Table S1. The replacements of the consensus phenylalanine (F^+2^) within the TorA signal peptide by a serine (F14S), an arginine (F14R), or an aspartate (F14D), resulting in plasmids pTorA(S^+2^)-MalE, pTorA(R^+2^)-MalE, and pTorA(D^+2^)-MalE) were done using the QuikChange® Site-Directed Mutagenesis Kit (Stratagene) with pTorA-MalE [16] as a template and primers RRF14Sfor53 and RRF14Srev53, RRF14Rfor53 and RRF14Rrev53, or RRF14Dfor53 and RRF14Drev53, respectively, according to the manufacturer’s instructions. Likewise, the combination of the TatC mutations K18E and L9F was constructed by using the same procedure with pHSG-TatABCE-KQS100 [13] as a template and primers K18E-for and K18E-rev, resulting in plasmid pRRD2-3. The single amino acid substitutions in the TatB protein, L9P or L9Q, were introduced into the different pHSG-TatABCE variants using a “crossover”-PCR method. For the construction of pHSG-TatABCE-RRD1-1 and pHSG-TatABCE-RRD3-1 respectively, a DNA fragment was amplified by the forward primer EP_TatABCE_For and a reverse primer (TatB_L9P_Ex_rev or TatB_L9Q_Ex_rev), which introduces the desired base exchange in the *tatB* gene with pHSG-TatABCE as a template. Next, a DNA fragment was amplified by using a forward primer (TatB_L9P_Ex_for or TatB_L9Q_Ex_for) which also carries the desired mutation in *tatB* and the primer EP_TatABCE_Rev with pHSG-TatABCE as a template. Both fragments were purified and used as a template in a cross-over PCR using primers EP-TatABCE_For and EP_TatABCE_Rev. The resulting PCR fragment was digested with *Eco*RI and *Sal*I and ligated into *Eco*RI/*Sal*I digested pHSG575. Plasmid pHSG-TatABCE-RRD1-3 was constructed by the same procedure using primers EP_TatABCE_For, TatB_L9P_Ex_rev, TatB_L9P_Ex_for, EP_TatABCE_Rev, and pHSG-TatABCE-KQS100 as the template. Likewise, plasmids pHSG-TatABCE-RRD3-3, pHSG-TatABCE-RRD6 and pHSG-TatABCE-RRD5 were constructed as described above, using primers EP_TatABCE_For, TatB_L9Q_Ex_rev, TatB_L9Q_Ex_for, EP_TatABCE_Rev, and plasmids pHSG-TatABCE-KQS100, pHSG-TatABCE-KQS105, or pHSG-TatABCE-RRD2-3 as a template, respectively. pTorA-GFP and pTorA(D^+2^)-GFP were constructed by amplifying the *torA-gfp* fusion gene from plasmid pCGTorA-GFP [40] via PCR using primers TorA_SP_fwd_Kpn1 and GFP_rev_EcoR1. The PCR product was digested with *Hpa*I and *Eco*RI and ligated in the *Hpa*I/*Eco*RI digested vector backbones of pTorA-MalE and pTorA(D^+2^)-MalE, respectively. *Hpa*I cuts within the coding region of the TorA signal peptide behind the codons of the Tat-consensus amino acids. *EcoR*I cuts behind the stop codon of *gfp* and *malE* respectively. In this way, *malE* was replaced by *gfp* and the coding regions of the TorA signal peptide and the TorA(D^+2^) signal peptide, respectively, were restored.

### Isolation of Tat Mutants

Plasmid pHSG-TatABCE-KQS100 [13] was mutagenized via error-prone PCR (ep-PCR) as described by Jaeger *et* *al.* [41]. A standard amplification reaction that resulted in a frequency of 1 to 7 point mutations per kilobase contained 20 ng of plasmid pHSG-TatABCE-KQS100 as a template, 5 pmol each of primers EP_TatABCE_For and EP_TatABCE_Rev, 6 mM MgCl_2_, 0.1−0.3 mM MnCl_2_, 0.2 mM dNTP’s, and 3 units of Taq polymerase (MBI Fermentas) in a total volume of 50 µl. After completion of the PCR, the amplified *tat* genes were cut with *Eco*RI and *Sal*I and ligated into *Eco*RI/*Sal*I-digested pHSG575. Subsequently, the ligation products were used to transform *E. coli* GSJ101 via electroporation. Approximately 10000 colonies were obtained, from which a pool of mutagenized pHSG-TatABCE-KQS100 plasmids was isolated. Small aliquots of this pool were transformed into GSJ101 (pTorA(D^+2^)-MalE) by electroporation. The transformed cells were plated on solid minimal medium containing 0.4% maltose and incubated at 37°C for up to 5 days. Some of the single mutant colonies that appeared on the selection plates were randomly picked. From these isolates, plasmid pHSG-TatABCE-KQS100 was isolated and retransformed into GSJ101 (pTorA(D^+2^)-MalE). Those pHSG-TatABCE-KQS100 plasmids that again restored growth of GSJ101 (pTorA(D^+2^)-MalE) on minimal medium agar plates containing 0.4% maltose were subsequently used for DNA sequence analysis and further functional characterizations.

### Miscellaneous Procedures

Fractionation of cells into a fraction containing the cytosol and membranes (C/M) and a periplasmic fraction (P) was done by using an EDTA-lysozyme spheroplasting method as described by Kreutzenbeck *et* *al.* [13]. Samples corresponding to an equal number of cells were subjected to sodium dodecyl sulfate-polyacrylamide gel electrophoresis (SDS-PAGE) and Western blotting using MalE-specific antibodies. As a control for the quality of the fractionation experiments, the subcellular localization of the cytoplasmic enzyme transaldolase B (TalB) was analyzed in parallel using TalB-specific antibodies. As expected, TalB was found exclusively in the C/M fractions of all cells examined (data not shown). Western blotting using anti-MalE and anti-TalB was performed by using the ECL Western blotting detection kit (GE Healthcare) according to the manufacturer’s instructions. The chemiluminescent protein bands were recorded using the Fujifilm LAS-3000 Mini CCD camera and image analyzing system together with the software AIDA 4.15 (Raytest). In our previous study [13], a different CCD camera (Fuji LAS-1000) and a different evaluation software (Aida 2.41; Raytest) were used for the semi-quantitative analysis of the protein bands. Due to this change of the hard- and software, the obtained numerical values of the semi-quantitative analysis data differ overall from those of our previous study. We noticed that the Fuji LAS-1000 model possessed a much lower dynamic range than the LAS-3000 system and it became obvious that we had previously significantly underestimated the amount of exported MalE in the positive control (which is always set as 100%). As a consequence, the previously reported numerical values for the relative export efficiencies of the mutant translocases were calculated too high. Importantly, however, the relative ranking of the intensities of the protein bands derived from wild-type and the various mutant strains is not affected by the changes in the recording system. Since Western blot quantification methods are semi-quantitative in nature, we would like to emphasize that the numerical values should not be taken as absolute values, but rather as a helpful means permitting a somewhat more descriptive comparison between different protein bands present on a given Western blot.

Preparation of membranes was performed as described previously [13]. Protein concentrations in the samples were determined by the method of Bradford [42]. SDS-PAGE and Western blotting using anti-TatA, anti-TatB, or anti-TatC antibodies were performed as described earlier [21]. Primary antibodies were detected and visualized by using an alkaline phosphatase conjugated second antibody together with nitro blue tetrazolium (NBT) and 5-bromo-4-chloro-3-indolyl phosphate (BCIP) as the substrates [43].

## Supporting Information

Figure S1 **Suppression of the TorA(D^+2^)-MalE export defect by KQS mutant translocases.** Cells were fractionated into a periplasmic (P) and a combined cytosol/membrane fraction (C/M) by EDTA-lysozyme spheroplasting. The samples were subjected to SDS-PAGE and immunoblotting using anti-MalE antibodies. The positive control was *E. coli* GSJ101 containing plasmids pTorA-MalE and pHSG-TatABCE (lane 1). The other samples correspond to GSJ101 containing plasmid pTorA(D^+2^)-MalE in addition to a pHSG-TatABCE plasmid that encodes one of the translocases indicated above the lanes. The nature of the signal peptide (SP) of the respective TorA-MalE precursors (wild-type (Wt) or containing the D^+2^ mutation (D+2)) and the TatB or TatC mutations present in the respective translocases are indicated in the box at the top of the figure. p, TorA-MalE/TorA(D^+2^)-MalE precursor in the C/M fraction; m, mature MalE in the P fraction; asterisk, TorA-MalE/TorA(D^+2^)-MalE degradation products in the C/M fraction. The phenotypes of the respective strains on MMM (-: no growth; +: slow growth; ++: growth) and MCM (P: pale; LR: light red/pink; R: red) agar plates are shown in the box at the bottom of the figure.
(TIF)

Figure S2 **Expression levels of TatA, TatB, and TatC proteins.** **A.** Membrane preparations corresponding to identical amounts of cells were subjected to SDS-PAGE and immunoblotting using specific antibodies directed against TatA (upper panel), TatB (middle panel), or TatC (lower panel). The samples correspond to *E. coli* GSJ101 containing plasmids pHSG575 (negative control, lane 1), pHSG-TatABCE (wild-type *tat* genes, lane 2), or the various pHSG-TatABCE plasmids expressing the mutant translocases (lanes 3–11) as indicated. **B**. The TatB (L9P) protein of mutant translocase RRD1-1 can be detected when the Western blot is over-exposed.
(TIF)

Figure S3 **The mutant Tat translocases are still able to handle the unaltered TorA-MalE precursor.** Cells were fractionated into a periplasmic (P) and a combined cytosol/membrane fraction (C/M) by EDTA-lysozyme spheroplasting. The samples were subjected to SDS-PAGE and immunoblotting using anti-MalE antibodies. The positive control was E. coli GSJ101 containing plasmids pTorA-MalE and pHSG-TatABCE (lanes 1). The negative control was E. coli GSJ101 containing plasmids pTorA(D^+2^)-MalE and pHSG-TatABCE (lanes 2). The other samples correspond to GSJ101 containing plasmid pTorA-MalE in addition to a pHSG-TatABCE plasmid that encodes one of the mutant translocases indicated above the lanes. Mutant translocases KQS100, KQS105, RRD2-3 (**A**), RRD1-3, RRD3-1, RRD3-3 (**B**), RRD1-1 (**C**), RRD6, RRD5 (**D**). The nature of the signal peptide (SP) of the respective TorA-MalE precursors (wild-type (Wt) or containing the D^+2^ mutation (D+2)) and the TatB and/or TatC mutations present in the respective translocases are indicated in the boxes at the top of the panels. p, TorA-MalE/TorA(D^+2^)-MalE precursor in the C/M fraction; m, mature MalE in the P fraction; asterisk, TorA-MalE/TorA(D^+2^)-MalE degradation products in the C/M fraction. All samples shown in the respective panels are derived from the same gel. However, in some cases lanes of the gels were removed to make the data easier to interpret.
(TIF)

Figure S4 **Subcellular localization of TorA(D^+2^)-GFP-derived polypeptides.** Cells were fractionated into a periplasmic (P) and a combined cytosol/membrane fraction (C/M) by EDTA-lysozyme spheroplasting. The samples were subjected to SDS-PAGE and immunoblotting using anti-GFP antibodies. The positive control was *E. coli* GSJ101 containing plasmids pTorA-GFP and pHSG-TatABCE (lane 1). The negative control, showing the export defect of TorA(D^+2^)-GFP in the presence of the wild-type Tat translocase, was E. coli GSJ101 containing plasmids pTorA(D^+2^)-GFP and pHSG-TatABCE (lane 2). All other samples correspond to GSJ101 containing plasmid pTorA(D^+2^)-GFP in addition to a pHSG-TatABCE plasmid that encodes one of the mutant translocases, as indicated above the lanes. The nature of the signal peptide (SP) of the respective TorA-GFP precursors (wild-type (Wt) or containing the D^+2^ mutation (D+2)) and the TatB and/or TatC mutations present in the respective translocases are indicated in the box at the top of the figure. p, TorA-GFP/TorA(D^+2^)-GFP precursor in the C/M fraction; m, mature GFP in the P fraction; asterisk, TorA-GFP/TorA(D^+2^)-GFP degradation products in the C/M fraction.
(TIF)

Table S1 **Primers used in this study.**
(DOCX)
