# Supplementary figures and images for: Genetic Evidence for a Tight Cooperation of TatB and TatC during Productive Recognition of Twin-Arginine (Tat) Signal Peptides in Escherichia coli
Source: PLoS One. 2012 Jun 26;7(6):e39867. doi: 10.1371/journal.pone.0039867 (PMC3383694; doi:10.1371/journal.pone.0039867)

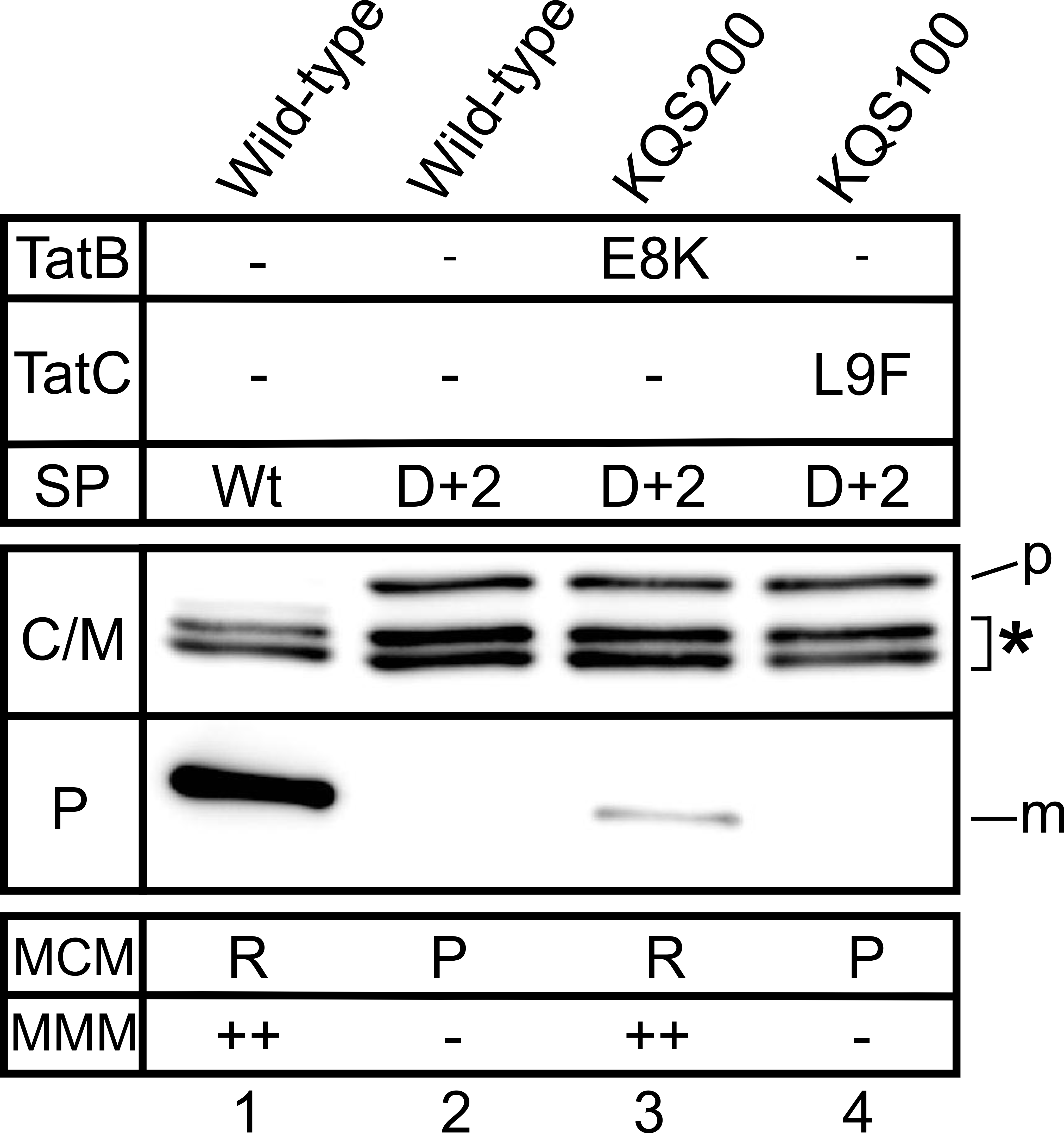

Supplement: Figure S1 — Suppression of the TorA(D+2)-MalE export defect by KQS mutant translocases. Cells were fractionated into a periplasmic (P) and a combined cytosol/membrane fraction (C/M) by EDTA-lysozyme spheroplasting. The samples were subjected to SDS-PAGE and immunoblotting using anti-MalE antibodies. The positive control was E. coli GSJ101 containing plasmids pTorA-MalE and pHSG-TatABCE (lane 1). The other samples correspond to GSJ101 containing plasmid pTorA(D+2)-MalE in addition to a pHSG-TatABCE plasmid that encodes one of the translocases indicated above the lanes. The nature of the signal peptide (SP) of the respective TorA-MalE precursors (wild-type (Wt) or containing the D+2 mutation (D+2)) and the TatB or TatC mutations present in the respective translocases are indicated in the box at the top of the figure. p, TorA-MalE/TorA(D+2)-MalE precursor in the C/M fraction; m, mature MalE in the P fraction; asterisk, TorA-MalE/TorA(D+2)-MalE degradation products in the C/M fraction. The phenotypes of the respective strains on MMM (-: no growth; +: slow growth; ++: growth) and MCM (P: pale; LR: light red/pink; R: red) agar plates are shown in the box at the bottom of the figure. (TIF) [file pone.0039867.s001.tif]

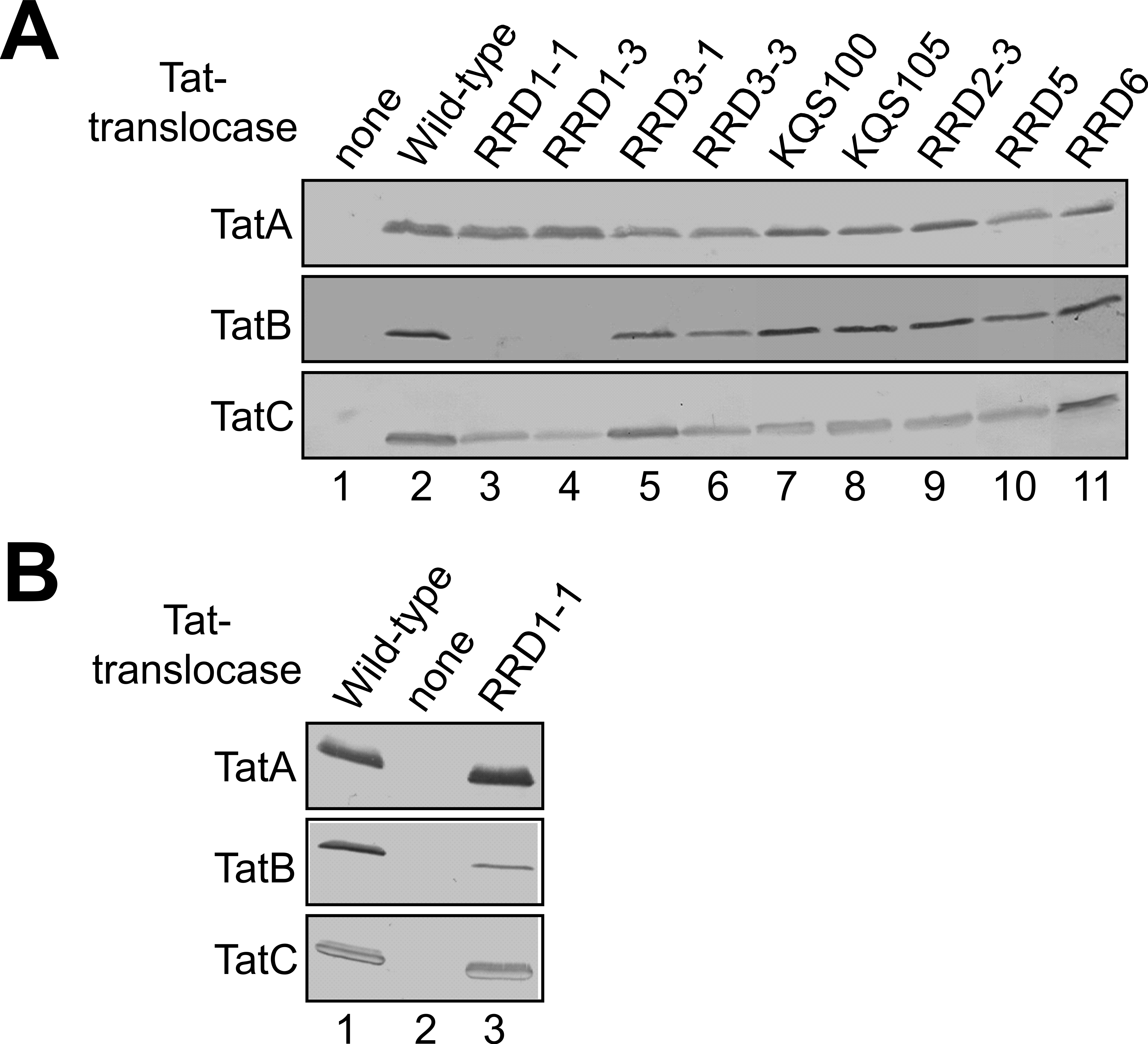

Supplement: Figure S2 — Expression levels of TatA, TatB, and TatC proteins. A. Membrane preparations corresponding to identical amounts of cells were subjected to SDS-PAGE and immunoblotting using specific antibodies directed against TatA (upper panel), TatB (middle panel), or TatC (lower panel). The samples correspond to E. coli GSJ101 containing plasmids pHSG575 (negative control, lane 1), pHSG-TatABCE (wild-type tat genes, lane 2), or the various pHSG-TatABCE plasmids expressing the mutant translocases (lanes 3–11) as indicated. B. The TatB (L9P) protein of mutant translocase RRD1-1 can be detected when the Western blot is over-exposed. (TIF) [file pone.0039867.s002.tif]

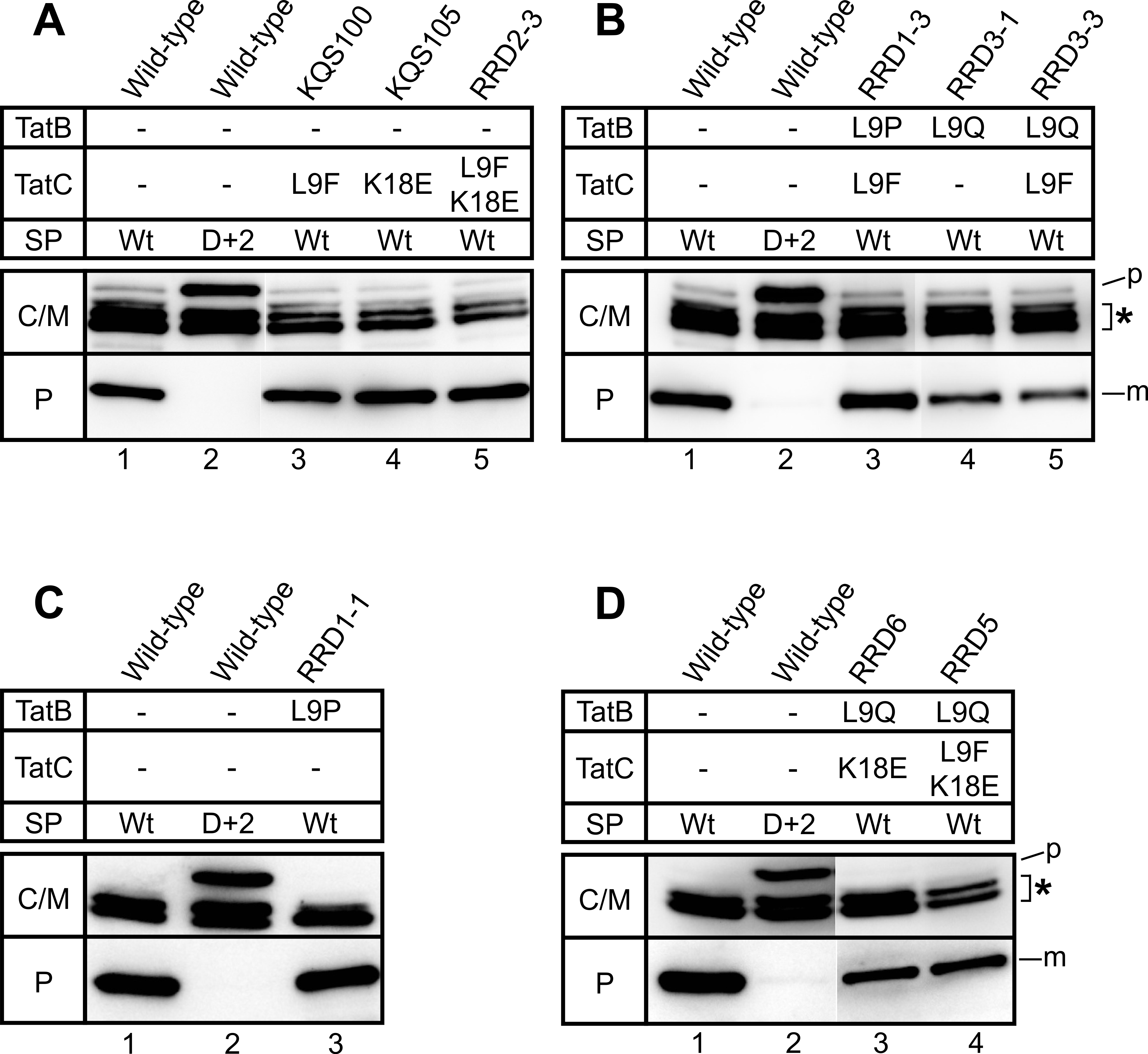

Supplement: Figure S3 — The mutant Tat translocases are still able to handle the unaltered TorA-MalE precursor. Cells were fractionated into a periplasmic (P) and a combined cytosol/membrane fraction (C/M) by EDTA-lysozyme spheroplasting. The samples were subjected to SDS-PAGE and immunoblotting using anti-MalE antibodies. The positive control was E. coli GSJ101 containing plasmids pTorA-MalE and pHSG-TatABCE (lanes 1). The negative control was E. coli GSJ101 containing plasmids pTorA(D+2)-MalE and pHSG-TatABCE (lanes 2). The other samples correspond to GSJ101 containing plasmid pTorA-MalE in addition to a pHSG-TatABCE plasmid that encodes one of the mutant translocases indicated above the lanes. Mutant translocases KQS100, KQS105, RRD2-3 (A), RRD1-3, RRD3-1, RRD3-3 (B), RRD1-1 (C), RRD6, RRD5 (D). The nature of the signal peptide (SP) of the respective TorA-MalE precursors (wild-type (Wt) or containing the D+2 mutation (D+2)) and the TatB and/or TatC mutations present in the respective translocases are indicated in the boxes at the top of the panels. p, TorA-MalE/TorA(D+2)-MalE precursor in the C/M fraction; m, mature MalE in the P fraction; asterisk, TorA-MalE/TorA(D+2)-MalE degradation products in the C/M fraction. All samples shown in the respective panels are derived from the same gel. However, in some cases lanes of the gels were removed to make the data easier to interpret. (TIF) [file pone.0039867.s003.tif]

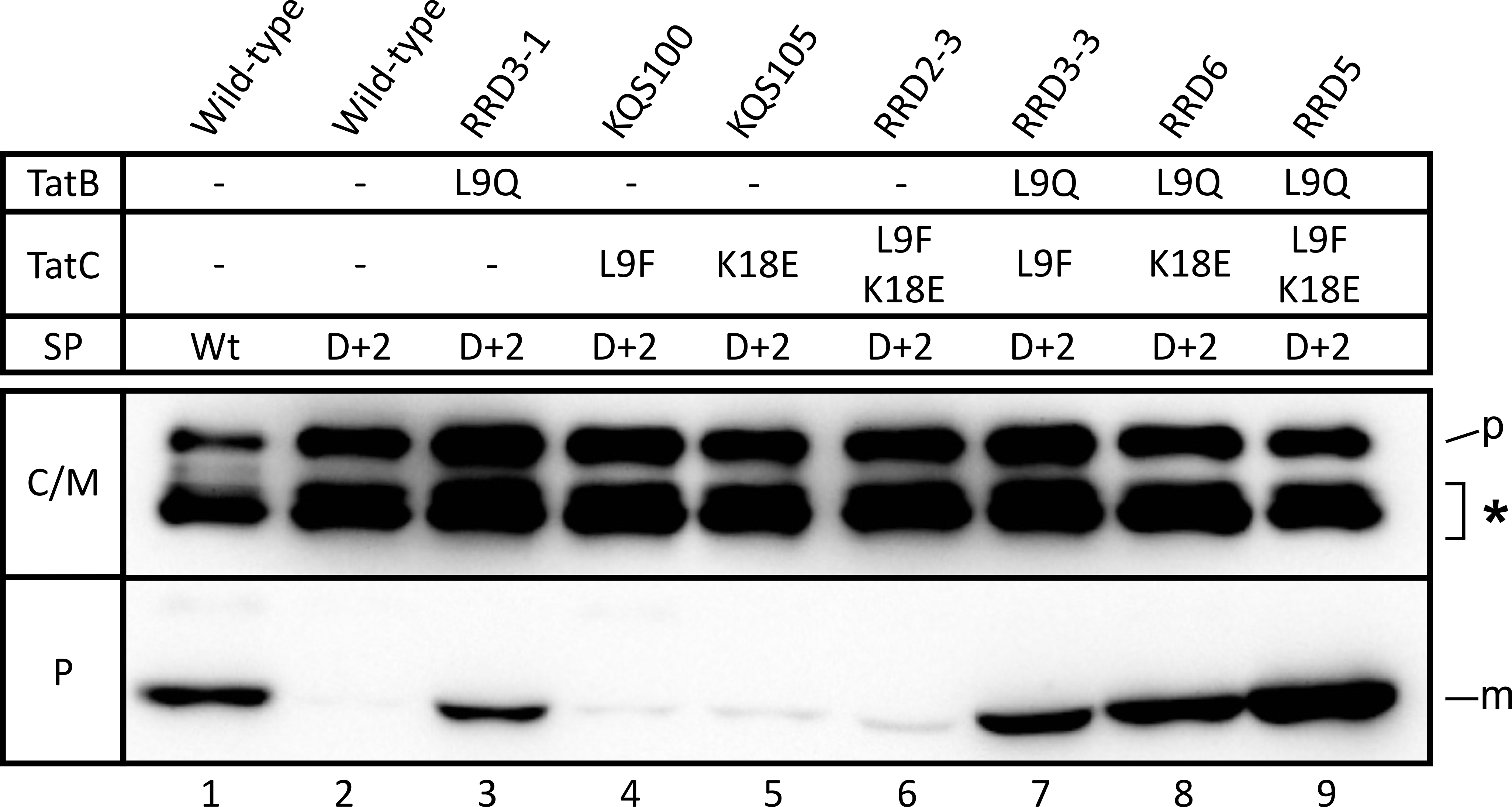

Supplement: Figure S4 — Subcellular localization of TorA(D+2)-GFP-derived polypeptides. Cells were fractionated into a periplasmic (P) and a combined cytosol/membrane fraction (C/M) by EDTA-lysozyme spheroplasting. The samples were subjected to SDS-PAGE and immunoblotting using anti-GFP antibodies. The positive control was E. coli GSJ101 containing plasmids pTorA-GFP and pHSG-TatABCE (lane 1). The negative control, showing the export defect of TorA(D+2)-GFP in the presence of the wild-type Tat translocase, was E. coli GSJ101 containing plasmids pTorA(D+2)-GFP and pHSG-TatABCE (lane 2). All other samples correspond to GSJ101 containing plasmid pTorA(D+2)-GFP in addition to a pHSG-TatABCE plasmid that encodes one of the mutant translocases, as indicated above the lanes. The nature of the signal peptide (SP) of the respective TorA-GFP precursors (wild-type (Wt) or containing the D+2 mutation (D+2)) and the TatB and/or TatC mutations present in the respective translocases are indicated in the box at the top of the figure. p, TorA-GFP/TorA(D+2)-GFP precursor in the C/M fraction; m, mature GFP in the P fraction; asterisk, TorA-GFP/TorA(D+2)-GFP degradation products in the C/M fraction. (TIF) [file pone.0039867.s004.tif]
