# Supplementary material for: Genetic Evidence for a Tight Cooperation of TatB and TatC during Productive Recognition of Twin-Arginine (Tat) Signal Peptides in Escherichia coli
Source: PLoS One. 2012 Jun 26;7(6):e39867. doi: 10.1371/journal.pone.0039867 (PMC3383694; doi:10.1371/journal.pone.0039867)
Supplement: Table S1 — Primers used in this study. (DOCX) [file pone.0039867.s005.docx]

**Table S1.** **Primers used in this study**

| Primer | Sequence (5’→3’) |
| --- | --- |
| RRF14Sfor53 | CTC TTT CAG GCA TCA CGT CGG CGT AGC CTG GCA CAA CTC GGC |
| RRF14Srev53 | GCC GAG TTG TGC CAG GCT ACG CCG ACG TGA TGC CTG AAA GAG |
| RRF14Rfor53 | CTC TTT CAG GCA TCA CGT CGG CGT CGC CTG GCA CAA CTC GGC |
| RRF14Rrev53 | GCC GAG TTG TGC CAG GCG ACG CCG ACG TGA TGC CTG AAA GAG |
| RRF14Dfor53 | CTC TTT CAG GCA TCA CGT CGG CGT GAT CTG GCA CAA CTC GGC |
| RRF14Drev53 | GCC GAG TTG TGC CAG ATC ACG CCG ACG TGA TGC CTG AAA GAG |
| K18E-for | GCA TCT GAT TGA GCT GCG TGA GCG TCT GCT GAA CTG C |
| K18E-rev | GCA GTT CAG CAG ACG CTC ACG CAG CTC AAT CAG ATG C |
| TatB_L9P_Ex_for | GGT TTT AGC GAA CCG CTA TTG GTG |
| TatB_L9P_Ex_rev | CAC CAA TAG CGG TTC GCT AAA ACC |
| TatB_L9Q_Ex_for | GGT TTT AGC GAA CAG CTA TTG GTG |
| TatB_L9Q_Ex_rev | CAC CAA TAG CTG TTC GCT AAA ACC |
| EP_TatABCE_For | CAG TGA GCG CAA CGC AAT TAA TG |
| EP_TatABCE_Rev | GCT GCG CAA CTG TTG GGA AG |
| TorA_SP_fwd_Kpn1 | GGGGTACCAAAGGAGGATAGATATGAACAATAACGATCTCTTTCAGGC |
| GFP_rev_EcoR1 | CGGAATTCTTATTTGTAGAGCTCATCCATGCC |
